# Ocular complaints and diagnoses in spaceflight

**DOI:** 10.1038/s41526-023-00335-7

**Published:** 2024-01-02

**Authors:** Elana Meer, Seanna R. Grob, Kris Lehnhardt, Aenor Sawyer

**Affiliations:** 1 Wayne and Gladys Valley Center for Vision, Department of Ophthalmology, University of California, San Francisco, CA USA; 2 University of California Space Health Program, San Francisco, CA USA; 3 Division of Oculofacial Plastic and Orbital Surgery, Wayne and Gladys Valley Center for Vision, Department of Ophthalmology, University of California, San Francisco, CA USA; 4 Exploration Medical Capability, NASA Johnson Space Center, Houston, TX USA; 5 Department of Orthopaedic Surgery, University of California, San Francisco, CA USA

**Keywords:** Eye manifestations, Eye abnormalities

## Abstract

The NASA human system risk board (HSRB) has long focused on trauma and acute medical illness as a key contributor to high level in-flight medical risk. However, ocular issues, trauma, and complaints during spaceflight are poorly characterized. In a retrospective case series, the NASA data from the life sciences data archieve (LSDA) and the lifetime surveillance of astronaught health (LSAH) was queried for eye related complaints and conditions in spaceflight across international space station (ISS) missions and space shuttle (STS) missions. The ISS dataset included missions from the year 2000 to 2020, and the STS dataset included missions from 1981 to 2011. Data were reviewed and segmented into categories of ocular complaints. 135 STS missions and 63 ISS missions were included in this analysis. Ocular events were only noted across 83 STS missions (61.5%) and 41 ISS missions (65.1%). Overall, the most common ocular complaints were eye irritation (*n* = 80, 33.1%), ocular foreign body or foreign body sensation (*n* = 55, 22.7%), dry eye syndromes (*n* = 38, 15.7%), epiphora or excessive tearing (*n* = 19, 7.85%). Of all ocular complaints or diagnoses, 9 (3.72%) were considered higher severity (keratitis, corneal ulcer, chemical exposure, and corneal abrasion). However, seemingly none required evacuation from mission. Improved depiction of ocular symptoms and diagnoses, and a more standard classification system and process to describe ocular symptoms, diagnoses, and treatments in space is crucial to provide more effective and comprehensive treatments.

## Introduction

The NASA human system risk board (HSRB) has long focused on trauma and acute medical illness as a key contributor to high level in-flight medical risk^1–4^. The NASA Engineering and Safety Center (NESC) and the Office of the Chief Health and Medical Officer (OCHMO) have sponsored multiple efforts with the goal of mitigating risks to human health on long-term expeditions beyond LEO (Moon, Mars, and beyond)^4^. Trauma is classified under “red” or “high” in-flight medical risk^4^, however, most research surrounding vision changes or symptoms in space focuses on spaceflight-associated neuro-ocular syndrome (SANS)^5,6^. Despite the emphasis on SANS, there are many potential sources of ocular injury or other ocular diagnoses that can be irritating, and even debilitating in space^3,4,6,7^. NASA evidence books report symptomatic complaints of eye foreign bodies, dry eyes, irritation, puffiness, and watering, as well as diagnoses of eye abrasions (from foreign bodies), eye chemical burns, and eye infections^3,7^. Similarly, Barratt’s Principles of Clinical Medicine for Spaceflight provides a review of key ophthalmic emergencies, focusing on corneal ulcers, corneal abrasions, and foreign bodies^6^. While these conditions may be the most commonly anticipated, they only represent three of a wide range of ocular concerns, necessitating a more extensive characterization of the ocular symptoms and diagnoses in spaceflight. Furthermore, there is limited publicly available data on ocular symptoms and diagnoses in spaceflight, making it difficult to consider and prioritize preventative, diagnostic, and treatment modalities for ophthalmic concerns on deep-space human spaceflight missions.

In this study, we aim to further characterize the ocular issues and complaints occurring in spaceflight. By investigating the reported ocular injuries for short and long-duration spaceflight, we hope to guide future efforts to improve diagnosis and treatment for ocular concerns in space.

## Methodology

### Data source and extraction

This retrospective cohort study utilizes deidentified data from astronauts evaluating all non-SANS-related ocular symptoms and diagnoses on all recorded international space station (ISS) and space shuttle (STS) missions. Due to the de-identified nature of the data report, this study was granted a not human subjects research (NHSR) determination IRB exemption from the NASA Institutional Review Board (eIRB STUDY00000467) as a quality improvement study with no risk or minimal risk to subjects and all secondary analysis performed on non-identifiable data. Patient consent for publication was not applicable as no identifiable information was obtained or included in the manuscript. Deidentified data was derived from the life sciences data archive (LSDA) and the lifetime surveillance of astronaut health (LSAH). LSDA is an active archive that provides information and data from 1961 (Mercury Project) through current flight analog studies (ISS) involving human, plant, and animal subjects, most of which are publicly available on this site or able to be requested for research^8^. The LSAH program explores and analyzes health risks associated with occupational exposures encountered by astronauts, examining acute, and chronic morbidity and mortality of astronauts^9^.

NASA data on astronaut non-SANS related ocular symptoms and diagnoses was queried with the following terms (encapsulated the most common ophthalmic conditions or complaints): corneal abrasion/abrasion, corneal ulcer, eye debris (small particles or foreign bodies noted in the eye), dry eyes, eye irritation, conjunctivitis (allergic, viral, and bacterial), keratitis (inflammation of the cornea, can be fungal, bacterial, etc.), meibomian gland dysfunction, keratopathy (e.g., filamentary keratopathy, exposure keratopathy, neurotrophic keratopathy, ultraviolet and keratopathy), PUffy Eyes/eyelids, Blepharitis, Foreign Body Into the Eye, Ocular Chemical/gas Exposure Or Incident, Ocular Chemical Burn, Ocular Infection (e.g., corneal infection, bacterial conjunctivitis, and endophthalmitis), contact lens related problems, lens dislocation, retinal/choroidal detachment, occlusion of the retinal artery, radiation injury, subconjunctival hemorrhage, globe disruption/open globe injury.

In line with the NASA NHSR determination, data was provided via subject-mission ID, not individual astronaut identification, with the occurrence of symptoms segmented by location on the ISS or STS. The ISS dataset contains data from ISS expeditions 1 to 63 from the year 2000 to 2020. There were 15 missions where eye events were not mentioned/recorded. Thus, this dataset contains eye events from 48 ISS missions. There were 41 astronaut crewmembers represented in the dataset, which means that if a crewmember flew twice, they are represented with a unique subject mission ID per mission.This STS dataset includes missions from STS 1 to 135 which totals 135 shuttle missions from the years 1981 to 2011. There were 52 missions where eye events were not mentioned/recorded. Thus, this dataset contains eye events from 83 Shuttle missions. There were 146 ocular events recorded out of 664 crewmembers (with and without ocular events) on all the STS missions. Ocular events were each recorded with a unique mission ID, which means that if a particular crewmember flew twice on a Shuttle mission, they are represented with a unique subject mission ID per mission. Similarly, if they had an ocular event on two different missions (even if it was a similar or related issue from a prior mission), each ocular event would be represented with a unique subject mission ID. STS crew missions were bucketed into mission lengths of 1–10 days and 11–20 days. ISS crew missions were bucketed into mission lengths of 1–150 days, 151–200 days, and 201–350 days. All missions included were less than 1 year.

### Statistical analysis

Data descriptions were first reviewed and eye diagnoses or symptoms were isolated. All analyses were completed on the astronaut level, repeat complaints were not counted multiple times. Descriptive analyses were used to describe key symptoms or diagnoses segmented by mission type, and mission length (days). Due to the lack of individual data and lack of standardization in the information provided about each complaint, statistical or predictive analyses could not be performed, with analyses focused on the presentation of symptomatic demographic data. Qualitative impressions of notes on ocular symptoms and treatments, when available, were also provided.

## Results

In total, 135 STS missions and 63 ISS missions were included in this analysis. Ocular events were only noted across 83 STS missions (61.5% of the total) and 41 ISS missions (65.1% of the total). In total, there were 242 ocular complaints noted across the 83 STS missions and 41 ISS missions, with all listed in Tables 1 and 2 segmented by mission type (ISS vs. STS) and mission length (days)

**Table 1:** Overall ocular symptoms and diagnoses segmented by mission type.

	Overall		Mission type				
	*n*	STS (*n*)	STS (% of total)	STS (% of total flights)	ISS (*n*)	ISS (% of total)	ISS (% of total flights)
Severe							
Keratitis	1	0	0.00%	0.00%	1	100.00%	1.59%
Eyelid laceration	2	0	0.00%	0.00%	2	100.00%	3.17%
Corneal abrasion	2	2	100.00%	1.48%	0	0.00%	0.00%
Chemical exposure	5	5	100.00%	3.70%	0	0.00%	0.00%
Trauma	1	1	100.00%	0.74%	0	0.00%	0.00%
Corneal ulcer	1	1	100.00%	0.74%	0	0.00%	0.00%
Non-Severe							
Erythema	4	0	0.00%	0.00%	4	100.00%	6.35%
Pruritis	3	1	33.33%	0.74%	2	66.67%	3.17%
Eye irritation	80	59	73.75%	43.70%	21	26.25%	33.33%
Dry eye syndromes (including keratoconjunctivitis Sicca, tear film insufficiency)	38	21	55.26%	15.56%	17	44.74%	26.98%
Eye pressure	3	1	33.33%	0.74%	2	66.67%	3.17%
Puffy eyes/eye fullness	6	4	66.67%	2.96%	2	33.33%	3.17%
Foreign bodies	55	48	87.27%	35.56%	7	12.73%	11.11%
Epiphora	19	15	78.95%	11.11%	4	21.05%	6.35%
Periorbital edema	7	2	28.57%	1.48%	5	71.43%	7.94%
Blepharitis	2	1	50.00%	0.74%	1	50.00%	1.59%
Photophobia	2	0	0.00%	0.00%	2	100.00%	3.17%
Tear film insufficiency	2	0	0.00%	0.00%	2	100.00%	3.17%
Pingueculitis	1	0	0.00%	0.00%	1	100.00%	1.59%
Lymphangioectasia	1	0	0.00%	0.00%	1	100.00%	1.59%
Hordeolum	2	1	50.00%	0.74%	1	50.00%	1.59%
Eye burning sensation	6	5	83.33%	3.70%	1	16.67%	1.59%
Eye pain	3	2	66.67%	1.48%	1	33.33%	1.59%
Conjunctival injection	10	5	50.00%	3.70%	5	50.00%	7.94%
Eye strain	1	1	100.00%	0.74%	0	0.00%	0.00%
Blurry vision	2	2	100.00%	1.48%	0	0.00%	0.00%
Bilateral subconjunctival hemorrhages	3	3	100.00%	2.22%	0	0.00%	0.00%
Subconjunctival hemorrhage	3	3	100.00%	2.22%	0	0.00%	0.00%
Retroorbital pain	1	1	100.00%	0.74%	0	0.00%	0.00%
Periorbital dermatitis	1	1	100.00%	0.74%	0	0.00%	0.00%

**Table 2:** Overall ocular symptoms and diagnoses segmented by mission length.

	Overall	Mission length (days)									
	n	1–10 (STS) (n)	1–10 (STS) (%)	11–20 (STS) (n)	11–20 (STS) (%)	0–150 (ISS) (n)	0–150 (ISS) (%)	151–200 (ISS) (n)	151–200 (ISS) (%)	201–350 (ISS) (n)	201–350 (ISS) (%)
Severe											
Keratitis	1	0	0.00%	0	0.00%	0	0.00%	1	100.00%	0	0.00%
Eyelid laceration	2	0	0.00%	0	0.00%	0	0.00%	1	50.00%	1	50.00%
Corneal abrasion	2	2	100.00%	0	0.00%	0	0.00%	0	0.00%	0	0.00%
Chemical exposure	5	2	40.00%	3	60.00%	0	0.00%	0	0.00%	0	0.00%
Trauma	1	1	100.00%	0	0.00%	0	0.00%	0	0.00%	0	0.00%
Corneal ulcer	1	0	0.00%	1	100.00%	0	0.00%	0	0.00%	0	0.00%
Non-Severe											
Erythema	4	0	0.00%	0	0.00%	3	75.00%	1	25.00%	0	0.00%
Pruritis	3	0	0.00%	1	33.33%	1	33.33%	1	33.33%	0	0.00%
Eye irritation	80	36	45.00%	23	28.75%	4	5.00%	9	11.25%	8	10.00%
Dry eye syndromes (including Keratoconjunctivitis sicca)	38	12	31.58%	9	23.68%	7	18.42%	10	26.32%	0	0.00%
Eye pressure	3	0	0.00%	1	33.33%	2	66.67%	0	0.00%	0	0.00%
Puffy eyes/ eye fullness	3	0	0.00%	2	66.67%	1	33.33%	0	0.00%	0	0.00%
Foreign bodies	55	22	40.00%	26	47.27%	0	0.00%	6	10.91%	1	1.82%
Epiphora	19	7	36.84%	8	42.11%	0	0.00%	3	15.79%	1	5.26%
Periorbital edema	7	0	0.00%	2	28.57%	0	0.00%	5	71.43%	0	0.00%
Blepharitis	2	0	0.00%	1	50.00%	0	0.00%	1	50.00%	0	0.00%
Photophobia	2	0	0.00%	0	0.00%	0	0.00%	1	50.00%	1	50.00%
Tear film insufficiency	2	0	0.00%	0	0.00%	0	0.00%	2	100.00%	0	0.00%
Pingueculitis	1	0	0.00%	0	0.00%	0	0.00%	1	100.00%	0	0.00%
Lymphangioectasia	1	0	0.00%	0	0.00%	0	0.00%	1	100.00%	0	0.00%
Hordeolum	2	0	0.00%	1	50.00%	0	0.00%	1	50.00%	0	0.00%
Eye burning sensation	9	5	55.56%	3	50.00%	0	0.00%	1	16.67%	0	0.00%
Eye Pain	3	0	0.00%	2	66.67%	0	0.00%	1	33.33%	0	0.00%
Conjunctival injection	10	3	30.00%	2	20.00%	0	0.00%	2	20.00%	3	30.00%
Eye strain	1	1	100.00%	0	0.00%	0	0.00%	0	0.00%	0	0.00%
Blurry vision	2	2	100.00%	0	0.00%	0	0.00%	0	0.00%	0	0.00%
Bilateral subconjunctival hemorrhages	3	2	66.67%	1	33.33%	0	0.00%	0	0.00%	0	0.00%
Subconjunctival hemorrhage	3	1	33.33%	2	66.67%	0	0.00%	0	0.00%	0	0.00%
Retroorbital pain	1	0	0.00%	1	100.00%	0	0.00%	0	0.00%	0	0.00%
Periorbital dermatitis	1	0	0.00%	1	100.00%	0	0.00%	0	0.00%	0	0.00%

Overall, the most common ocular complaints were eye irritation (*n* = 80, 33.1% of total complaints), ocular foreign body or foreign body sensation (*n* = 55, 22.7% of total complaints), dry eye syndromes (*n* = 38, 15.7% of total complaints), epiphora or excessive tearing (*n* = 19, 7.85% of total complaints) (Table 1). Of all ocular complaints or diagnoses, 9 (3.72% of the total) were considered higher severity (keratitis, corneal ulcer, chemical exposure, and corneal abrasion). However, none required evacuation from a mission.

### Ocular symptoms and diagnoses by mission type

The most common eye issues on STS missions were eye irritation symptoms (73.8% of total), dry eye syndromes (55.6% of total), puffy eyes/eye fullness (66.7% of total), eye burning sensation (88.9% of total), eye pain (66.7% of total) (Table 1). All symptomatic complaints or diagnoses of eye strain, blurry vision, subconjunctival hemorrhage, corneal abrasion, chemical exposure, retroorbital pain, trauma, corneal ulcer, and periorbital dermatitis occurred on STS (Table 1). Of all ocular complaints of STS (*n* = 188), the most common symptoms were eye irritation (31.4% of all STS complaints), dry eye syndromes (11.2% of all STS complaints), ocular foreign body or foreign body sensation (25.5% of all STS complaints), and epiphora or excessive tearing (7.98% of STS complaints) (Table 1).

Of all ocular complaints, the majority of pruritis symptoms (66.7% of total), eye pressure (66.7% of total), and periorbital edema (71.4% of total), as well as all photophobia, keratitis, eyelid laceration, and erythema presented on ISS (Table 1). The most common complaints on ISS were eye irritation (26.3% of all ISS complaints) and dry eye syndromes (21.3% of total ISS complaints) (Table 1).

### Ocular symptoms and diagnoses by mission length

Overall ocular complaints segmented by mission length are demonstrated in Table 2. Of note, the number of complaints in each subset of mission length was too small for any statistical analysis, therefore, we only present demographic data without any statistically tested or significant associations or conclusions. When comparing 1–10 day and 11–20 day buckets on STS mission, eye irritation symptoms (45% of total), burning sensation (55.6% of total), subconjunctival hemorrhages (66.7% of total), and eye strain and blurry vision (100% of total) most commonly occurred during 1–10 days of STS missions (Table 2). Puffy eyes/eye fullness (66.7% of total), ocular foreign bodies or foreign body sensation (40% of total), and eye pain (66.7% of total) more commonly occurred during 11–20 days of STS missions. On ISS, erythema (75% of total) and eye pressure (66.7% of total) occurred most commonly on 0–150 days on mission, keratitis (100% of total) on 151–200 days on mission, and photophobia and eyelid lacerations on 201–350 days on mission (50% of total) (Table 2).

### Ocular symptoms and diagnoses during extravehicular activities (EVA)

Of all ocular complaints, 14 (5.2% of total complaints) occurred during or immediately after spacewalks (extravehicular activities or EVA). Ten of these cases (71.4% of EVA complaints) involved complaints of eye irritation from particulates, with two of these also reporting conjunctival injection. These irritation symptoms were reported to resolve without intervention after the end of the spacewalk when the astronauts had removed their helmets. Three ocular complaints (21.4% of EVA complaints) involved eye burning thought to occur secondary to the antifog substance sprayed into the visor/helmet. One astronaut (7.1% of EVA complaints) experienced congestion and puffy eyes, which resolved with pseudoephedrine treatment on return from EVA.

### Common causes of ocular complaints

While the cause of symptoms was not consistently annotated, a qualitative assessment for common mentions of symptom causes was performed. Among the most common complaints of eye irritation, foreign body sensation, and dry eye syndromes, the reported causes included particulates in the air, sweat, irritants in spacesuits during EVAs, higher carbon dioxide levels, food particle dust (tortilla dust, shrimp dust, etc), lint particles, anti-fog treatment, salt crystals, lithium hydroxide dust (substance used for the removal of carbon dioxide), omniprep from attaching biomedical electrodes, and aluminum particles in the air.

### Common treatments for ocular complaints

While treatments were not consistently annotated, a qualitative assessment for common mentions of treatments was performed. Among the most common complaints of eye irritation, foreign body sensation, and dry eye syndromes, reported treatments included saline solution rinses as needed, artificial tears as needed, fexofenadine (for concomitant allergic symptoms), pseudoephedrine (for concomitant congestion symptoms), and tetryzoline drops as needed.

## Discussion

In this study, we present self-reported data on ocular symptoms and diagnoses on ISS and STS missions. The most common symptoms across all mission types and mission lengths were eye irritation, ocular foreign body or foreign body sensation, dry eye syndromes and symptoms, and tearing. The majority of these symptoms, along with puffy eyes/eye fullness and congestion, and all symptoms and diagnoses of eye strain, blurry vision, subconjunctival hemorrhage, corneal abrasion, chemical exposure, retroorbital pain, trauma, corneal ulcer, and periorbital dermatitis occurred on STS. Out of total STS missions, STS missions also had a higher ratio of presentation of eye irritation, ocular foreign body, epiphora, subconjunctival hemorrhage, corneal abrasion, chemical exposure, retroorbital pain, and trauma compared to ISS. ISS did have a greater ratio of total ISS missions presenting with dry eye syndromes compared to STS. The number of cases was too small to make any claims of differential prevalence across mission types or mission lengths. Certain conditions trended towards presenting during shorter mission lengths (eye irritation, burning sensation), however, there were no substantial differences in presentation segmented by mission length and statistical analysis was not possible given the sample size. There may have been a trend of more severe symptoms for longer missions (e.g., keratitis and eyelid lacerations), however, further analysis by mission length was limited by sample size. Interestingly, our results did not suggest that severe conditions were more frequent on longer trips, however, they were more common on STS missions. This could be due to vehicle differences, better training or procedures, or more rigorous schedules on STS, however, further investigation is needed to better elucidate this observation.

While the most common presentations and symptomatic complaints were not considered emergent, irritation, dry eye syndromes, and foreign body sensations can be extremely bothersome, and even debilitating. Even nuisance conditions such as foreign body sensations and dry eye syndromes can affect performance, which will have a mission impact. In the terrestrial literature, the increased prevalence of depression and anxiety in patients with persistent dry eye syndromes, concomitant ocular irritation, and foreign body sensation has been widely reported, further increasing the importance of recognizing and managing these conditions in spaceflight^10–18^.

The most common symptoms of irritation, foreign body sensation, and dry eye occurred in response to different particulates and chemical exposure (carbon dioxide, anti-fog treatment, and lithium hydroxide). Given known exposures in spaceflight, these common causes of eye complaints are in line with past reports^6^. Crew members may be exposed to dust through EVAs (requiring a spacewalk)^19^, which can be secondary to the planetary surface work itself if suit breakdown or debris trapped in the suit leads to persistent irritation. Particulate exposure may also occur on return from EVAs, or while changing control life support system filters increasing the risk for ocular exposure and injury^20–23^. Apollo missions also expose astronauts to celestial dust, which may gain entry into the vehicle on return to EVAs or during maintenance procedures. Celestial dust is also predisposed to causing irritation due to its particular characteristics, with lunar dust containing sharp edges and Mars dust containing perchlorate irritants^20–23^.

However, while celestial and air particulates were noted, a common cause of irritation included food particulates, such as dust from tortilla or shrimp packages, which has not been previously reported. In addition, irritation was a common complaint during EVA missions, in which astronauts spend hours in a pressurized suit with eyes exposed to whatever particles and chemicals are within the suit to keep it functional. For example, many astronauts experienced irritation specifically to the antifog treatment (soap) used to ensure that they could see clearly on EVAs, suggesting that it might have an opposite effect to that intended. Of note, astronauts cannot wear goggles under their helmets to protect from these exposures, only glasses if necessary.

The most common treatments for these conditions included different artificial tears and rinse solutions. While analysis of treatments used among these cases is limited due to missing information and incomplete notes, it is clear that artificial tears and saline rinses were frequently used to treat irritative symptoms, dry eyes, and foreign body sensations. However, it is unclear which specific artificial tear solutions are being used. More specifically, there were multiple mentions of tetryzoline or tetryzoline “red” drops, which is classically avoided by ophthalmologists and optometrists as it has been shown to produce rebound hyperemia and persistent irritative symptoms if used regularly^24^. Instead, preservative-free solutions would be recommended due to decreased ocular toxicity, especially for artificial tear solution usage multiple times a day^25,26^. In addition, preservative solutions may even disrupt the corneal epithelial barrier and predispose astronauts to further ocular irritation, foreign body sensation, and corneal abrasion^26,27^. While preservative-free solutions would undoubtedly be preferred by ophthalmologists as a treatment for dry eye, foreign body sensation, and irritative symptoms, it is important to note that strict mass and volume allocations on spacecraft may pose a challenge. Preservative-free solutions typically are packaged in single containers with a shorter shelf-life (discard ~24 h after opening) when compared to preserved solutions (discard ~weeks to months after opening), therefore, it may be difficult to have sufficient artificial tears for both preventative management and symptomatic treatment^28,29^. Given the frequency of irritative symptoms occurring during/after EVA, cargo loading, maintenance activities, and eating, it may be advised to wear eye protection more frequently to further protect from celestial (on Apollo missions), chemical, and food particles in the air. Goggles may be lightweight, flexible, and slip-free for comfort^30^. However, further iterations on goggles provided on ISS and STS, as well as on EVAs that fit comfortably under the helmets, may be necessary to encourage greater compliance. It is also important to discuss potential onboard medications and equipment that could ameliorate the more severe complications experienced on a mission, such as corneal ulcers. For examination of the cornea, a small portable slit lamp could be useful for examination along with the ability to take anterior segment photos to send back to Earth for consultation if needed. Fluorescein strips in coordination with a blue light (either on a portable slit lamp or separately) are useful in the analysis of the corneal surface for epithelial defects in the case of an ulcer or an abrasion. Common ophthalmic antibiotic drops include fluoroquinolones, such as moxifloxacin, which are beneficial in cases of ulcers or epithelial defects to prevent infection. Ideally, ulcers that are large or threatening the visual axis are cultured so treatment can be directed toward the specific microbe, but this may not be possible in space. If there is concern for corneal perforation, corneal glue could be utilized in the area of concern for ulcer perforation. However, corneal glue may require significant expertise to successfully utilize and therefore if there is concern for ulcer perforation, the best course of management may be rapid evacuation for surgical management on earth. Golf club spud instruments or 27–30-gauge needles may be useful for metal foreign bodies embedded in the cornea, which may occur in the setting of engineering tasks on board. However, these additional diagnostic and treatment modalities may not be possible given strict mass and volume allocations.

This study suffers from a number of limitations. First, due to the deidentified nature of this data, presented with individual mission IDs rather than individual astronaut identifiers, it is difficult to assess ocular complaints, conditions, and incidences on the individual astronaut level. Second, we are limited by the number of subjects included in this study, and therefore it is difficult to characterize differences in presentation across mission type and mission length. Third, the data extracted was from notes on self-reported symptoms from astronauts and terrestrial medical support personnel (none of whom is a trained eyecare provider). As a result, there is a lack of consistency in the language used to describe symptomatology and diagnoses, as well as an incomplete presentation of data. Similarly, certain complaints such as blurry vision are multifold and could be associated with multiple ocular diagnoses and etiologies, even including SANS. Finally, while some notes mentioned treatments used and timeline of presentation, others did not. More specifically, as this data did not include how specific symptoms or diagnoses were determined or managed, we do not know the severity or details of the conditions. This is especially challenging for conditions with a range of severity, such as eyelid lacerations and/or corneal abrasion or foreign body. Therefore, further details of these symptoms and diagnoses would be useful. Additionally, the data did not provide any relative impact to crew performance, which would be helpful to triage the importance of diagnosis and treatment. Efforts could focus on those complaints with greater effects on crew performance. Therefore, future studies would benefit from access to comprehensive clinical data, including medical practitioner notes, assessments, management plans, and overall prognosis and time to recovery with the proposed management plans. It is also important to note that no associations could be drawn based on the length of microgravity exposure as the reported symptoms and diagnosis are few, limiting statistical analysis. Future studies may benefit from further analysis of symptoms segmented by mission length.

The lack of consistency in terminology to describe ocular signs, symptoms, and diagnoses is a particularly important consideration and makes the interpretation of this data challenging. For example, a note could have mentioned eye redness, however, this could mean a variety of findings or diagnoses including conjunctival injection from irritation, allergies, foreign body, hemorrhage or inflammation or periorbital erythema from a stye, blepharitis, allergies, dermatitis or others. This challenge in describing symptoms, or ophthalmic taxonomy, does not only apply to spaceflight but also to global health and teleophthalmology, in which clear descriptors are incredibly important in the absence of in-person eye exams performed by trained specialists^31^. In addition, the descriptors found in this dataset did not allow for quantification of symptoms, making it difficult to characterize the severity or duration of an ocular condition, which is integral information for providing effective and comprehensive treatment. Therefore, this data further highlights the need for a more descriptive (qualitative and quantitative) taxonomy for characterizing ophthalmologic symptoms and diagnoses in spaceflight and other remote settings. While the authors can only make suggestions based on the limited data available, a classification system in which symptoms are categorized in accordance with a standardized ophthalmic review of systems may be helpful. Such symptom descriptions may be segmented into visual symptoms (vision changes, blurry vision, flashes or floaters, black curtain falling over vision, visual field defect), eye sensation and appearance symptoms (eye pain, redness, irritation, discharge, itchiness, dry eyes/foreign body sensation), extraocular movements (double vision, restriction with extraocular movements, pain with extraocular movements), and any systemic symptoms associated with vision loss (jaw claudication, scalp tenderness, fever, fatigue, weight loss, etc). Next, it will be integral to have a standardized clinical examination associated with the symptoms, which would include visual acuity, intraocular pressure, confrontational visual fields, extraocular motility photos or video, anterior segment photography and posterior segment photography. These standard and regular examinations could help further classify the eye symptoms and also the common or more vision-threatening issues in space and allow for better management or preparation of treatment protocols in space. Additionally, existing triage algorithms exist for assisting in triage of layperson description of ocular complaints, and may be incorporated into decision algorithms when synchronous access to medical personnel is not possible^32^.

In conclusion, this study presents the self-reported data on ocular injuries and ocular symptomatic complaints across 135 STS and 63 ISS missions. Eye irritation, dry eye symptoms, and foreign body sensations are frequently reported issues in spaceflight. While this study did not have enough subjects to explore differences in the overall severity of symptomatic complaints segmented by length of mission, a trend may exist towards more serious conditions such as keratitis and eyelid lacerations presenting on longer missions. Since the occurrence of severe ocular conditions on a deep space exploration mission could have serious consequences for both the astronauts on board as well as the mission itself, further work may be needed to plan for treatment and care of more serious eye conditions that may occur on these missions. Finally, improved data collected on ocular symptoms and diagnoses, and a standardized classification system and process to describe ocular symptoms, diagnoses, and treatments in space would enable the development of more effective and comprehensive treatments for spaceflight.

## Data Availability

Deidentified data was derived from the Life Sciences Data Archive (LSDA) and the Lifetime Surveillance of Astronaut Health (LSAH), however, unfortunately, this data was not permitted to be publically available. No codes were utilized to analyze the data, only descriptive statistics were used.

## Code availability

Deidentified data was derived from the Life Sciences Data Archive (LSDA) and the Lifetime Surveillance of Astronaut Health (LSAH), however, unfortunately, this data was not permitted to be publically available. No codes were utilized to analyze the data, only descriptive statistics were used.
